# The relaxation of non-pregnant rat myometrium by resveratrol with participation of the NO–cGMP pathway

**DOI:** 10.1186/2050-6511-13-S1-A24

**Published:** 2012-09-17

**Authors:** Radmila B Novaković, Dragana D Protić, Nebojša V Radunović, Vladimir I Kanjuh, Ljiljana C Gojković-Bukarica

**Affiliations:** 1 Institute of Pharmacology, Clinical Pharmacology and Toxicology, Medical Faculty, University of Belgrade, 11129 Belgrade, Serbia; 2 Institute of Gynecology and Obstetrics, Clinical Center of Serbia, 11129 Belgrade, Serbia; 3 Serbian Academy of Sciences and Arts, 11129 Belgrade, Serbia

## Background

Resveratrol (RSV) is a phytoalexin produced by grapevines. The benefit of resveratrol to health is widely reported. Resveratrol has been found to promote vascular relaxation but its mechanism of action is unclear. Data about an influence of RSV on the contractility of smooth muscles of the uterus are not available. It has been claimed that NO promotes uterine relaxation by the elevation of cyclic GMP. It is generally accepted that NO increases the intracellular cGMP concentration through activation of solubile guanylate cyclase (sGC). The aim of our study were to investigate the effects of RSV on the contractility of rat uterus and to investigate the involvement of the NO–cGMP pathway in the relaxant effect of RSV on spontaneous rhythmic contractions (SRC) and phasic contractions provoked by oxytocin.

## Methods

Uterine strips were obtained from virgin female Wistar rats in oestrus. Strips were mounted into organ bath for recording isometric tension in Krebs-Ringer solution. Experiments followed a multiple curve design. In order to test the involvement of the NO–cGMP pathway in the mechanism of action of RSV, a methylene blue (methylthionine chloride; MB) inhibitor of sGC was used.

## Results

RSV induced a concentration-dependent relaxation of SRC with pD_2_ = 4.53 and E_max_ of 89% and of contractions provoked by oxytocin with pD_2_ = 4.66 and E_max_ of 94% (p < 0.05). MB (10 µM) antagonized the response to RSV in both oxytocin-induced contractions and SRC with pD_2_ = 4.31 and pD_2_ = 4.24, E_max_ = 76% and E_max_ = 67%, respectively.

## Conclusions

RSV is a uterine relaxant and can be used in tocolysis. The antagonism by MB of the RSV effect suggests that NO–cGMP pathways are involved in RSV action on the contractions of rat uterus. However, the relative resistance to MB of resveratrol’s effects at concentrations higher than 30 μM indicated an additional mechanism of action.

